# Health literacy-related knowledge, attitude, perceived barriers, and practice among primary care doctors in Malaysia

**DOI:** 10.1038/s41598-023-47242-1

**Published:** 2023-11-13

**Authors:** Mohamed-Syarif Mohamed-Yassin, Aqil M. Daher, Anis Safura Ramli, Nabilah Farhana Ramli, Noorhida Baharudin

**Affiliations:** 1 Department of Primary Care Medicine, Faculty of Medicine, Universiti Teknologi MARA, Sungai Buloh, Selangor, Malaysia; 2 Department of Community Medicine, School of Medicine, International Medical University, Kuala Lumpur, Malaysia; 3 College of Health and Medical Techniques, Almaaqal University, Basrah, Iraq; 4 Hospital Melaka, Ministry of Health, Melaka, Malaysia; 5 Institute of Pathology, Laboratory and Forensic Medicine (I-PPerForM), Universiti Teknologi MARA, Sungai Buloh, Selangor, Malaysia

**Keywords:** Health policy, Patient education, Public health

## Abstract

This study aimed to assess the health literacy (HL) related knowledge, attitude, perceived barriers, and practice among primary care doctors (PCDs) in Malaysia, and to determine the factors associated with HL-related practice. A cross-sectional study was conducted using an online questionnaire. Sociodemographic and work-related details were collected. HL-related knowledge, attitude, perceived barriers, and practice were assessed. Descriptive and inferential analyses using linear regression were performed. 373 PCDs were included in the study with a mean (SD) age of 37.9 (8.1) years old. The mean (SD) HL-related knowledge, attitude, and practice scores were 6.89 (1.27), 36.33 (7.04), and 30.14 (4.7), respectively. 90.9% of the participants had good HL-related knowledge scores, and 89.5% had positive HL-related attitude. More than 80% of participants found that “time constraint to implement health literacy screening” and “lack of human resources to administer HL screening tools in their settings” were among the barriers for them to implement HL practices. PCDs of Chinese and other ethnicities had lower HL-related practice scores compared to those of Malay ethnicity (adjusted b = − 1.74; 95% CI − 2.93, − 0.54, and − 2.94; 95% CI − 5.27, − 0.60, respectively). PCDs who had heard of the term “health literacy” were associated with higher HL-related practice scores (adjusted b = 2.32; 95% CI 1.17, 3.47). Age (adjusted b = 0.10; 95% CI 0.04, 0.16) had significant linear positive relationship with HL-related practice. In conclusion, the HL-related knowledge, attitude, and practice among PCDs in Malaysia were at an acceptable level. Along with educating PCDs on HL, the perceived barriers identified need to be addressed to improve the HL-related practice and ultimately patient care.

## Introduction

Health literacy (HL) is defined as a person’s “knowledge, motivation and competency to access, understand, appraise, and apply health information, in order to make judgements and take decisions in everyday life concerning health care, disease prevention and health promotion, to maintain or improve quality of life during the life course”^1^. Prevalence of limited HL is surprisingly high even in developed countries including Australia and the United States^2,3^. According to the 2015 European Health Literacy Survey (HLS-EU), the prevalence of limited HL was between 29 to 62% among eight European countries^4^. In Malaysia, the National Health and Morbidity Survey (NHMS) 2019 reported that 35% of adults above 18 years old had limited health literacy^5^.

Numerous studies have reported poor outcomes in people with limited HL. These include poor medication adherence^6–8^, increased emergency care use and hospitalization risk^6,9^, and inability to obtain suitable health services and preventative health screening^10^. In view of this evidence, the importance of HL has been recognized in health care and within the public health context. European policy documents such as the European Commission White Paper (Together for Health)^11^, the World Health Organization (WHO) Regional Office for Europe’s Health 2020 strategy^12^, and the WHO’s Health literacy: the solid facts publication have all incorporated the concept of HL^13^. In 2016, at the WHO’s Ninth Global Conference on Health Promotion in Shanghai, member countries including Malaysia endorsed the Shanghai declaration. Signatories of this declaration committed to recognize HL as a critical determinant of health and invest in its development. They also pledged to develop, implement and monitor intersectoral national and local strategies for strengthening HL in all populations and all educational settings^14^.

In Malaysia, primary care has been recognized as the spine to the health service delivery^15^. In the public sector alone, there were 2892 primary care clinics in 2021^16^, which catered for 64% of all outpatient services, while private clinics covered the remaining 36%^17^. A study among type 2 diabetes mellitus patients in primary care clinics in Perak, Malaysia found that the prevalence of limited health literacy was 65.3%^18^. Another study among Malaysian elderly primary care patients in Selangor found that 19.1% had limited HL^19^. This is in comparison to a study conducted among two primary health care centres in Lithuania, which reported that 40.6% of patients had problematic health literacy^20^.

Doctors working in the primary care setting are often the first port of call for most patients seeking medical treatment or advice in Malaysia. Therefore, primary care doctors (PCDs) could play a pivotal role in identifying patients with limited HL and address this issue accordingly. However, studies have reported that many healthcare providers are not aware that limited HL has a significant impact on patients and the healthcare system^21^. It has also been reported that many healthcare providers lack the knowledge and skill necessary to identify and intervene for patients with limited HL^22,23^. To date, there is no published research on PCDs’ HL-related knowledge, attitude, practice, and perceived barriers in Malaysia. Hence, this study aimed to determine the HL-related knowledge, attitude, practice, and perceived barriers among PCDs in Malaysia. This study also aimed to determine the factors associated with HL-related practice in this population.

## Materials & methods

### Study design, setting and participants

This was a cross-sectional study conducted among PCDs in Malaysia using an online questionnaire via *Google Forms*™. The inclusion criteria for this study were PCDs who were fully registered with the Malaysian Medical Council, were working in a primary care setting at the time of the study, had worked at least six months in primary care in Malaysia, and were able to understand written English. Those who were no longer actively practising in a primary care setting in Malaysia for the six months preceding the study or worked in primary care only as locums were excluded from the study.

PCDs were broadly categorized into those with postgraduate qualification in family medicine and those without. Postgraduate qualifications in family medicine that are recognized by the National Specialist Register in Malaysia are the Diploma of Family Medicine (DFM), Advanced Training in Family Medicine (ATFM), Fellowship of the Royal Australian College of General Practitioners (FRACGP), Master of Medicine (Family Medicine), and Member of the Royal College of General Practitioners (MRCGP) UK. Those who held any of these qualifications were defined as having “postgraduate qualification in family medicine”. Those who held only a basic medical degree were categorized as having “no postgraduate qualification in family medicine”. PCDs were also divided into their respective designations. Family Medicine Specialists were defined as those with FRACGP, Master of Medicine (Family Medicine) and MRCGP UK. Medical officers were defined as those who worked in government health clinics or university primary care clinics who have not qualified as a Family Medicine Specialist. On the other hand, those who worked in private clinics and have not qualified as a Family Medicine Specialist were defined as general practitioners.

### Study tool

There were five sections to the study questionnaire: sociodemographic and work-related characteristics, HL-related knowledge, HL-related attitudes, HL-related practice, and perceived barriers to HL practice. Section 1 collected basic demographic details (age, sex, ethnicity) and work-related characteristics (type of clinic, number of patients seen in a day, name of medical school, years of working experience as a doctor and in primary care, postgraduate qualifications in family medicine, whether has heard of the term of HL).

Sections 2 to 5 were adapted from a questionnaire by Rajah et al.^24^. Section 2 contained eight items on HL-related knowledge, with “true”, “false” or “do not know” options. This section assessed the definition of functional HL and low HL, assessment of HL, benefits of HL to healthcare professionals, and effects of low HL. One point was awarded for each “true” answer, while no points were awarded for “false” and “do not know” answers. The correct answers were summed to obtain a total score. The range of scores was between 0 to 8, with scores ≥ 6 indicating good knowledge, and < 6 indicating poor knowledge^24^. Section 3 consisted of nine items assessing HL-related attitude. They were 5-point Likert-type scale responses (1 = strongly disagree, 2 = disagree, 3 = neutral, 4 = agree, 5 = strongly agree). The total scores were calculated, ranging between 9 to 45, with scores ≥ 32 indicating positive attitude, and < 32 indicating negative attitude^24^. For Sect. 4, eight items on the practice of HL-related communication strategies were assessed, using 5-point Likert scale responses (never = 1, seldom = 2, sometimes = 3, often = 4, always = 5). These scores were then summed, with a range of scores between 8 to 40. Higher scores indicated better HL-related practices. Ten items on HL-related perceived barriers were assessed in Sect. 5, requiring five-point Likert-type scale responses (1 = strongly disagree, 2 = disagree, 3 = neutral, 4 = agree, 5 = strongly agree). Options 1, 2 and 3 were then categorized as “did not identify as a barrier”, while scores 4 and 5 were categorized as “identified as a barrier”.

The original questionnaire had been validated in health professionals including doctors working in Penang Hospital, Malaysia^24^. The internal consistency reliability for knowledge of HL questions was 0.76 (Kuder-Richardson 20). The Cronbach alpha coefficients for attitude, perceived barriers, and practice of HL communications were 0.78, 0.91, and 0.87 respectively. Permission to adapt into the online *Google Forms*™ version and use this questionnaire had been obtained from the author.

### Sample size determination

Sample size was calculated based on the objectives of this study. First, using Statulator’s sample size calculator for estimating a single mean (https://statulator.com/SampleSize/ss1M.html), with a SD of 3.86 based on Rajah et al.’s study, with 95% confidence intervals, and precision of 0.5, the required sample size was 232^24^. The sample size for multivariable linear regression analysis was also determined. We followed Bujang et al.’s recommendation of a sample size of at least 300^25^.

### Recruitment, sampling method, and data collection

Participant recruitment was conducted from August 2021 to November 2021. The committee members of three of the largest primary care doctors’ organizations, which were the Academy of Family Physicians Malaysia (AFPM), Malaysians Family Medicine Specialists’ Association (FMSA), and the Malaysian Primary Care Network (MPCN) were approached. The objectives of the study were explained to them. All three associations then forwarded a short description of the study with an invitation link via email and/or social media messages to all their members. Reminder email and/or social media messages were sent after 1, 2 and 4-week interval from the initial invitation.

For those who clicked on the invitation link, they would be directed to the first page of the *Google Forms*™. This page contained the study information. Informed consent was obtained from all participants. Participants who consented to participate were required to click on a button labelled “Next” to proceed to the next section. Then, the inclusion and exclusion criteria were checked by the participants ticking a series of boxes. Those who fulfilled both criteria will then proceed to the actual questionnaire. This study utilized the convenient purposive sampling method until the target sample size of at least 300 participants was achieved.

### Data entry and statistical analysis

Data were entered, checked, and analyzed using the IBM® Statistical Package for Social Sciences (SPSS) software version 28 (IBM Corp., Armonk, NY, USA). For categorical data, descriptive statistics were presented as frequencies and percentages, while for continuous data, mean with standard deviations (SD) were presented. Next, univariate analysis utilizing simple linear regression was conducted to determine the factors associated with HL-related practice. Factors with a ***P***-value of < 0.05 were then included in the multivariable linear regression analysis, to adjust for the confounders. For this analysis, ***P***-values of < 0.05 were considered statistically significant. The results were presented as regression coefficients, with 95% confidence intervals (CI).

### Ethics approval

The study was conducted in accordance with the Declaration of Helsinki and approved by the Universiti Teknologi MARA’s Institutional Research Ethics Committee (Ref: REC/12/2020 [MR/478]).

## Results

### Participants’ characteristics and HL-related knowledge, attitude, and practice scores

**Figure 1:**
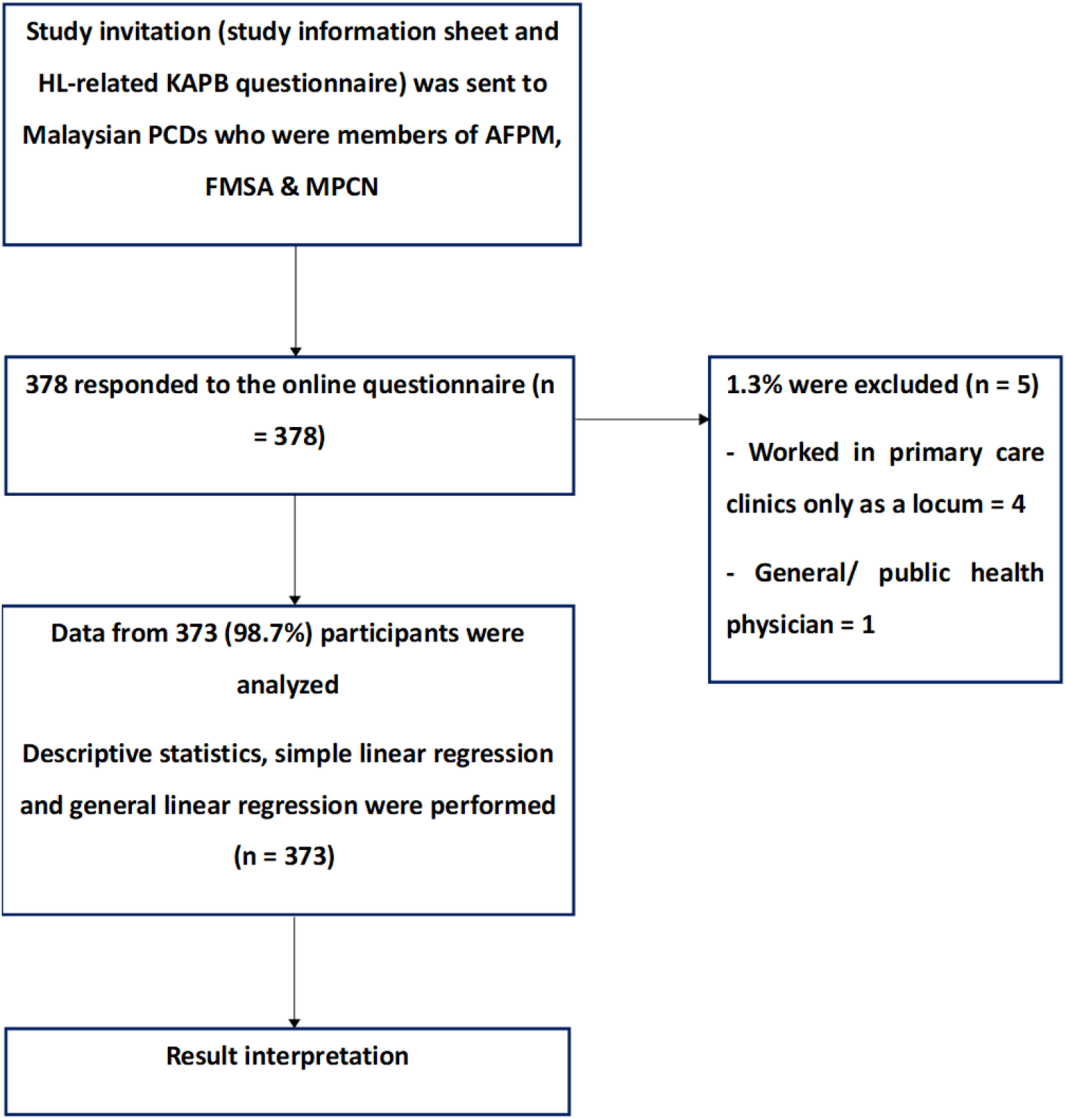
Study flowchart.

A total of 378 *Google Forms*™ responses were received. Five (1.3%) were excluded as four respondents worked in primary care clinics only as locums and one respondent did not work in a primary care setting (Fig. 1). From the remaining 373 PCDs who were included in the analysis, the mean age (SD) was 37.9 (8.1) years old, with a majority of them being females (76.1%), Malays (64.9%), and worked in government health clinics (75.4%) (Table 1). The mean (SD) HL-related knowledge, attitude, and practice scores were 6.89 (1.27), 36.33 (7.04), and 30.14 (4.70), respectively. 90.9% of the participants had good HL-related knowledge scores, and 89.5% had positive HL-related attitude.

**Table 1:** Participants’ characteristics and HL-related knowledge, attitude, and practice scores (n = 373).

Characteristic	n (%)	Mean (SD)
Age (years)		37.9 (8.1)
Less than 30	34 (9.1)	
30 to 39	247 (66.2)	
40 and above	92 (24.7)	
Gender		
Male	89 (23.9)	
Female	284 (76.1)	
Ethnicity		
Malay	242 (64.9)	
Chinese	70 (18.8)	
Indian	46 (12.3)	
Others	15 (4)	
Postgraduate qualification in family medicine		
No	159 (42.6)	
Yes	214 (57.4)	
Designation		
Medical Officer	221 (59.2)	
Family medicine specialist	130 (34.9)	
General practitioner	22 (5.9)	
Place of work		
Government Health Clinic	281 (75.4)	
Private Clinic	37 (9.9)	
University Primary Care Clinic	55 (14.7)	
Experience working as a doctor (years)		12.6 (7.6)
< 10	191 (51.2)	
≥ 10	182 (48.8)	
Experience working in primary care (years)		8.6 (6.7)
< 10	256 (68.6)	
≥ 10	117 (31.4)	
Location of medical school		
Malaysia	219 (58.7)	
Overseas	154 (41.3)	
Heard of the term “health literacy”		
No	74 (19.8)	
Yes	299 (80.2)	
HL-related knowledge score		6.89 (1.27)
Poor	34 (9.1)	
Good	339 (90.9)	
HL-related attitude score		36.33 (7.04)
Negative	39 (10.5)	
Positive	334 (89.5)	
Practice of HL score		30.14 (4.70)

### Perceived barriers related to health literacy practices among primary care doctors in Malaysia

More than 80% of participants found that “time constraint to implement health literacy screening” and “lack of human resources to administer health literacy screening tools in their settings” were among the barriers for them to implement health literacy practices. A majority (> 70%) also found “lack of easy-to-use tools or instruments to identify patients with limited health literacy”, and “lack of organizational resources to implement health literacy strategies” as their barriers (Table 2).

**Table 2:** Perceived barriers related to health literacy practices among primary care doctors in Malaysia (n = 373).

Perceived barrier	n (%)	
	No	Yes
Time constraint to implement health literacy screening	62 (16.6)	311 (83.4)
Lack of human resources to administer health literacy screening tools in the clinical setting	72 (19.3)	301 (80.7)
Lack of easy-to-use tools or instruments to identify patients with limited health literacy	100 (26.8)	273 (73.2)
Lack of organizational resources to implement health literacy strategies	101 (27.1)	272 (72.9)
Lack of organization/ leadership commitment to promote health literacy	118 (31.6)	255 (68.4)
Lack of patient commitment towards health literacy strategies provided	139 (37.3)	234 (62.7)
Lack of awareness about ways by which patients hide their limited health literacy	150 (40.2)	223 (59.8)
Lack of knowledge on health literacy and its consequences	159 (42.6)	214 (57.4)
Lack of patient co-operation to assess their health literacy	163 (43.7)	210 (56.3)
Lack of interest about enhancing health literacy	227 (60.9)	146 (39.1)

### Factors associated with health literacy related practice

Table 3 shows the findings from the simple linear regression analyses performed. Seven out of 12 independent variables were significant (*P* < 0.05) from these analyses.

**Table 3:** Association of sociodemographic, work-related factors, HL-related knowledge and attitude, with HL-related practice using simple linear regression.

Characteristics	Crude b (95% CI)	*P*-value
*Age (years)*	0.11 (0.05, 0.16)	** < 0.001**
Gender		
Male	Ref	0.176
Female	0.77 (− 0.35, 1.90)	
Ethnicity		
Malay	Ref	
Chinese	− 1.99 (− 3.23, − 0.76)	**0.002**
Indian	− 0.90 (− 2.36, 0.56)	0.227
Others	− 3.08 (− 5.50, − 0.66)	**0.013**
Postgraduate qualification		
No	Ref	
Yes	1.49 (0.54, 2.45)	**0.002**
Designation		
Medical Officer	Ref	
Family medicine specialist	1.68 (0.67, 2.69)	**0.001**
General practitioner	0.18 (− 1.86, 2.23)	0.859
Place of practice		
Government health clinic	Ref	
Private clinic	− 0.25 (− 1.86, 1.37)	0.765
University primary care clinic	0.73 (− 0.64, 2.09)	0.297
Experience working as a doctor (years)	0.12 (0.06, 0.18)	** < 0.001**
Experience working in primary care (years)	0.13 (0.06, 0.20)	** < 0.001**
Location of medical school		
Malaysia	Ref	
Overseas	0.50 (− 0.47, 1.48)	0.309
Heard of the term “health literacy”		
No	Ref	
Yes	2.70 (1.53, 3.87)	** < 0.001**
HL-related knowledge score		
Poor	Ref	
Good	1.42 (− 0.24, 3.08)	0.094
HL-related attitude score		
Negative	Ref	
Positive	1.56 (0.00, 3.12)	0.05

Ref-reference group; **Emboldened**: *P* < 0.05.

Multivariable linear regression analyses were performed incorporating these seven variables (Table 4). Stepwise, forward, and backward methods were performed. Based on the principle of parsimony, the model using the backward method with four significant factors, and R^2^ of 0.11 was chosen. There were no significant biologically meaningful interactions found, and no multicollinearity. All assumptions were checked (linearity, independence, normality of response variable, equal variances & homoscedasticity, & independent numerical variable linearity) and were fulfilled. There were no outliers.

**Table 4:** Factors associated with health literacy related practice from multivariable linear regression.

Characteristics	Adjusted b (95% CI)	*P*-value
*Age (years)*	0.10 (0.04, 0.16)	** < 0.001**
Ethnicity		
Malay	Ref	
Chinese	− 1.74 (− 2.93, − 0.54)	**0.005**
Indian	− 1.20 (− 2.63, 0.23)	0.099
Others	− 2.94 (− 5.27, − 0.60)	**0.014**
Heard of the term “health literacy”		
No	Ref	
Yes	2.32 (1.17, 3.47)	** < 0.001**

Backward multivariable linear regression method was applied. Model assumptions were fulfilled.

No multicollinearity was detected. There were no significant biologically meaningful interactions found. Coefficient of determination (R^2^) = 0.11. Ref-reference group; **Emboldened: ***P* < 0.05.

Four factors were associated with health literacy related practice (Table 4). These were age, Chinese ethnicity, other ethnicity, and heard of the term “health literacy”. PCDs of Chinese and other ethnicities had lower HL-related practice scores compared to those of Malay ethnicity with adjusted b of − 1.74; 95% CI − 2.93, − 0.54, and − 2.94; 95% CI − 5.27, − 0.60, respectively. PCDs who had heard of the term “health literacy” were associated with higher HL-related practice scores (adjusted b = 2.32; 95% CI 1.17, 3.47). Age (adjusted b = 0.10; 95% CI 0.04, 0.16) had significant linear positive relationship with HL-related practice whereby an increase in age by one year would increase the HL-related practice score by 0.1.

## Discussion

This study found that the majority of PCDs in Malaysia had good HL-related knowledge (90.9%). This contrasted with a study on healthcare workers in Malaysian public hospitals in Penang which reported that 60.8% of physicians had good HL-related knowledge^24^. This could be contributed by the fact that almost 80% of the PCDs in the current study compared to only 43.7% of these physicians had heard of the term HL. Several other studies worldwide also found that health care professionals had insufficient knowledge on HL^26–28^.

Our study found that 89.5% of Malaysian PCDs had positive HL-related attitude. This was higher compared to the physicians in Malaysian public hospitals where only 60.5% had positive HL-related attitude^24^. This study involving healthcare providers in public hospitals in Penang also reported that the proportion of pharmacists and nurses who had positive attitudes toward HL were even lower, at 52.7% and 41.1%, respectively. The authors proposed that these health care providers may have placed more importance on other patient care issues that have a noticeable impact to patient care, given the heavy workload in public hospitals^24^.

The current study found that the main perceived barriers related to health literacy practices were time constraint to implement health literacy screening (83.4%), lack of human resources to administer health literacy screening tools in the clinical setting (80.7%), and lack of easy-to-use tools or instruments to identify patients with limited health literacy (73.2%). These were consistent with several other international studies^24,29,30^ These identified barriers are understandable given the known heavy workload in the Malaysian public sector as the majority of the participants for this study worked in government clinics^16^.

HL-related practice mean (SD) score was 30.14 (4.70). This was higher compared to the another Malaysian study (range 25.02 – 27.29)^24^. The difference could be due to the participants in that study included nurses and pharmacists along with physicians. Our multivariable linear regression analysis found that age had a significant positive linear relationship with HL-related practice. This finding is consistent with Rajah et al.’s study where they found those aged 41 years and older had the highest mean HL-related practice score^24^. It is interesting to note that their study also found practitioners with ten or more years of service had higher HL-related practice score compared to those with less than ten years of service. This contrasted with our multivariable analysis which did not find either experience working as a doctor (in years) or experience working in primary care (in years) to be significantly associated with HL-related practice.

However, similar to the study by Rajah et al.^24^, we found that PCDs who had heard of the term “health literacy” were associated with higher HL-related practice scores. This emphasizes the need to incorporate HL into current undergraduate and postgraduate medical education, as well as in continuous professional development programs for all health care professionals.

### Strengths and limitations of this study

To the best of the authors’ knowledge, this is the first study to assess health literacy-related knowledge, attitude, perceived barriers, and practice among PCDs in Malaysia. However, the study findings need to be interpreted in the context of several limitations. Firstly, as with all cross-sectional studies, the findings can only show association but causal relationship between the variables studied and the HL-related practice. Secondly, the online recruitment method may introduce selection bias. To limit this, extensive efforts were made to ensure that the study invitations reached as many Malaysian PCDs as possible and repeated reminders were also sent.

### Implications on clinical practice and future research

The findings of this study suggest that efforts should be made to reduce or eliminate the perceived barriers to HL-related practice. The recently presented Health White Paper by the Ministry of Health Malaysia has pledged to optimize the primary health care delivery in this country, while enhancing the collaboration between public–private health care institutions, as well as reviewing the intake of healthcare workers in the public sector based on the numbers, profession, skill-mix, and maldistribution between regions^15^. This will hopefully help to reduce the workload of healthcare professionals especially doctors which may then allow them to communicate with their patients using HL-appropriate techniques. Next, medical schools and specialist colleges should also incorporate education on HL in their curriculum and continuous medical education programs so that future doctors learn about HL and its effects on patient care. Finally, the R^2^ for the multivariable linear regression model was 0.11. This suggests that collectively the independent variables in the final regression model could explain 11% of the variance in HL-related practice score. Hence, future research needs to explore and include other possible independent variables that may contribute to HL-related practice.

## Conclusions

The HL-related knowledge, attitude, and practice among PCDs in Malaysia were at an acceptable level. However, there were several perceived barriers to HL-related practice including time constraints and lack of human resources to implement HL screening. Along with educating PCDs on HL, these barriers need to be addressed to improve the HL-related practice and ultimately patient care.

## Data availability 

The data that support the findings of this study will be made available to others, from the publication date, by emailing the corresponding author.
